# Editorial: Nanocarbons: Basics and Advanced Applications

**DOI:** 10.3389/fchem.2021.657941

**Published:** 2021-03-16

**Authors:** Lipiao Bao, Yun-Peng Xie, Long Qie

**Affiliations:** ^1^ Department of Chemistry and Pharmacy & Joint Institute of Advanced Materials and Processes (ZMP), Friedrich-Alexander University of Erlangen-Nürnberg, Erlangen, Germany; ^2^ State Key Laboratory of Materials Processing and Die & Mould Technology, School of Materials Science and Engineering, Huazhong University of Science and Technology, Wuhan, China; ^3^ Institute of New Energy for Vehicles, School of Materials Science and Engineering, Tongji University, Shanghai, China

**Keywords:** nanocarbons, fullerene, graphene, carbon fiber, water purification

Carbon materials like soot, charcoal, graphite, and diamond have been known to human being even in prehistory and play a vital role in the development of human civilization. The world of carbon was further extended by the recent discovery of new nanoscale carbon members like fullerene and graphene. Due to the distinct dimensionalities and atomic structures featured by these nanocarbons, extraordinary physio-chemical properties have been expected, of which some have already been experimentally demonstrated including solubility, strength, electrical/thermal conductivity, and so on. Research into this direction and the further advanced applications are currently one of the center topics in both the academic and industrial communities.

In this special issue, we have provided some new understanding into nanocarbons, covering diverse aspects from the fundamental structures and chemistry of fullerenes to advanced applications of graphene and carbon fibers. Wang and coworkers presented a comprehensive review over the structural analysis of giant fullerenes composed of 100 or more cage carbons. Because of the large cage size and the resulting low yield, poor solubility and numerous possibilities of cage isomerization, the synthesis, purification and structural determination of these cages are quite challenging. This review has not only summarized the current progress in this topic but also pointed out the future development of giant fullerenes. In a study of the chemistry of nanocarbons, Pan’s group studied a radical reaction of monometallofullerene Y@C_2v_(9)-C_82_ with N-arylbenzamidine catalyzed by silver carbonate. This reaction is highly regioselective affording only one monoadduct with the addend linked to a specific [5,6]-bond closed to the endohedral Y atom, as a result of the unique cage geometry showing the highest pyramidalization angle at the addition sites according to the computational analysis. Beyond these fundamental aspects, Wang’s group fabricated a modified carbon fiber material (Ag/CCF/H-EP) used as an electrically responsive shape memory composite. By varying the content of Ag/CCF in the composites, the effects of Ag/CCF on the mechanical, electrical and thermal properties of the composites were thoroughly studied. Liu and coworkers reviewed the current application progress of the magnetic graphene materials for water purification. They presented an exhaustive overview of the reported examples, including the preparation methods, separation effect and underlying mechanisms.

As the guest editors of this exciting issue, we’d like to thank all authors and reviewers for their valuable contributions. This issue demonstrates the broad interest in nanocarbons, from basic structures and chemistry, to advanced applications in various fields. We hope that these articles presented in this topic issue will stimulate more interests into this exciting nano-world of carbon.

